# Prognostic Value of Left and right ventricular deformation strain analysis on Acute Cellular rejection in Heart Transplant recipients: A 6-year outcome study

**DOI:** 10.1007/s10554-022-02586-z

**Published:** 2022-08-08

**Authors:** Robert Chamberlain, Natalie F.A. Edwards, Samantha N. Doyle, Yee Weng Wong, Gregory M. Scalia, Surendran Sabapathy, Jonathan Chan

**Affiliations:** 1 Department of Cardiology, The Prince Charles Hospital, Brisbane, Australia; 2 School of Medicine, Menzies Health Institute Queensland, Griffith University, Gold Coast, Australia; 3 School of Medicine, University of Queensland, Brisbane, Australia; 4 The Prince Charles Hospital, Department of Cardiology, Rode Road, 4032 Chermside, Queensland Australia

**Keywords:** Strain imaging, Speckle-tracking, Allograft rejection, Prognosis.

## Abstract

**Purpose:**

Two-dimensional (2D) strain analysis is a sensitive method for detecting myocardial dysfunction in acute cellular rejection (ACR) from post-transplant complications. This study aims to evaluate the utility of novel left (LV) and right ventricular (RV) strain parameters for prognostic risk stratification associated with ACR burden at 1-year post transplantation.

**Methods:**

128 Heart transplant patients, assessed between 2012 and 2018, underwent transthoracic echocardiography and endomyocardial biopsy. 2D strain analysis was performed and history of rejection burden was assessed and grouped according to ACR burden at 1-year post transplantation. The primary endpoint was all-cause mortality at 6-years follow up.

**Results:**

21 patients met primary the endpoint. Multivariate analysis of 6-year all-cause mortality showed LV global longitudinal strain (LV GLS) (Hazard Ratio [HR] = 1.21, CI = 1.06–1.49), LV early diastolic strain rate (LV ESr) (HR = 1.31, CI = 1.12–1.54), RV GLS (HR = 1.12, CI = 1.02–1.25) and RV ESr (HR = 1.26, CI = 1.12–1.47) were significant predictors of outcome. Univariate analysis also showed LV GLS, LV ESr, RV GLS and RV ESr were significant predictors of outcome. Optimal cut-off for predicting 6-year mortality for LV GLS by receive operator characteristic was 15.5% (sensitivity: 92%, specificity: 79%). Significant reductions (p < 0.05) in LV GLS, RV GLS and LV and RV ESr between rejection groups were seen.

**Conclusions:**

Non-invasive LV and RV strain parameters are predictors of mortality in post-transplant patient with ACR. LV GLS and LV ESr are superior to other strain and conventional echo parameters.

## Introduction

Acute cellular rejection ACR is considered one of the main causes of death in the first-year post heart transplant, along with acute graft failure and infection, but is most frequently experienced during the first 3–6 months. Primarily a histopathological diagnosis by endomyocardial biopsy, ACR can be classified according to severity based on standardised gradient from mild (grade 1R) to severe (grade 3R). Although such classification allows a dichotomised decision to augment immunosuppression intensity or otherwise, a single timepoint grading fails to capture the longitudinal and cumulative nature of the immune mediated process of ACR. Furthermore, studies have highlighted the prognostic implication of recurrent mild ACR in the pathogenesis of cardiac allograft vasculopathy (CAV)[1–3] and long-term clinical outcomes. Therefore, additional non-invasive clinical markers that may complement routine invasive surveillance biopsies may prove valuable in further risk stratification of heart transplant recipients based on burden of recurrent cellular rejection.

Transthoracic echocardiography is the first line non-invasive imaging modality for surveillance of allograft function post-transplant, and commonly includes 2D and Doppler assessment of bi-ventricular size and function to help determine changes associated with post-transplant complications. However, traditional 2D and Doppler parameters such as left ventricular ejection fraction (LV EF) and trans-mitral diastolic Doppler parameters are not advocated in the long-term assessment of allograft function in the setting of ACR or CAV[4–7].

Global Longitudinal Strain (GLS) derived from 2D echocardiographic studies has been shown to be a sensitive tool for detection of subtle changes in longitudinal myocardial function, secondary to fibrosis and oedema in the sub-endocardial tissue layer in many cardiac disease states[8, 9]. 2D strain has shown to be more sensitive at detecting ACR and CAV in-light of LV EF being within guideline recommended normal limits[10–13]. LV GLS has been reported to have prognostic value in the first-year post-transplant and recent findings show LV GLS and RV strain to be reduced in transplanted hearts[14–16]. The medium and long-term impact of repeated ACR on LV and RV strain parameters however remain unknown. Thus, the aim of this study was to examine the impact of repeated episodes of ACR on LV and RV function measured by 2D strain analysis, and to determine prognostic risk stratification of 2D strain parameters analysed at 1-year post transplantation associated with ACR burden during the first year after transplantation.

## Methods

### Study cohort

Data was drawn from a pool of 322 heart transplant patients referred for transthoracic echocardiography at a tertiary hospital in Queensland, Australia between 2012 and 2018. There were 149 patients referred who had known transplant vasculopathy and were therefore excluded. Of the 173 patients remaining, 1 patient was transplanted at another institution, 18 had incomplete information of previous rejection and 26 patients were excluded because of poor image quality. The final cohort comprised of 128 transplant patients (mean age 49 ± 15 years) who were followed up during the first-year post-transplant with regular endomyocardial biopsies. Patients with LV EF < 50%, moderate or greater mitral regurgitation, moderate or greater aortic stenosis, aortic valve replacement and patients who were pacing dependent were also excluded from the study. Biopsies were performed regularly throughout the first-year post transplantation according to local hospital protocol using a standard surveillance biopsy regimen. Biopsies were performed weekly in weeks 1–6 and then during weeks 8, 12, 18, and 26 with additional biopsies performed during steroid weaning or when ACR was clinically suspected. The biopsy technique included using the internal jugular or femoral vein and specimens were examined by a cardiac pathologist. ACR was diagnosed via biopsy using the 2005 International Society of Heart and Lung Transplantation (ISHLT) grading system[17].

A rejection score (RS) was used to classify patients into one of three groups as previously described [18, 19].

- RS group 1 (mild): <50% of biopsies with grade 1R ACR and no rejection higher than 1R.
- RS group 2 (moderate): One episode of ≥ 2R ACR or > 50% of biopsies with 1R.
- RS group 3 (severe): More than one episode of ≥ 2R ACR.

The number of episodes of ACR allowed for determination of grouping with ACR episodes occurring prior to endpoints occurring. Routine transthoracic echocardiography with 2D strain analysis was performed at 1-year post-transplantation. A statewide electronic medical record was used to review patients included in the study for the primary endpoint of all-cause mortality 6-years post day of transplantation. Secondary endpoints of cardiovascular morbidity 6-years post transplantation consisting of: development of CAV determined by coronary angiography as per ISHLT guidelines[20], coronary stenoses requiring percutaneous coronary intervention and/or coronary artery bypass surgery (CABG), hospitalisation with symptoms of heart failure, new arrhythmia, de-novo regional wall motion abnormalities on stress echocardiography and new episodes of ACR or antibody mediated rejection were also reviewed using the statewide electronic medical record.

### Echocardiography

Full, comprehensive transthoracic echocardiograms were performed on each patient as part of the routine clinical care at 1-year post transplantation using one of two commercially available ultrasound systems: iE33 digital ultrasound system with either an S5-1 or X5-1 transducer (Philips, Andover, MA, USA) or a GE Vivid E9 digital ultrasound system with a M5S 3.5 MHz transducer (GE Medical, Milwaukee, WI, USA) with subsequent 2D strain analysis performed by R.C. M-mode, 2D imaging and Doppler imaging were performed from parasternal, apical, subcostal, and suprasternal windows. The apical images were performed in the left lateral decubitus position with images optimised to visualise the entire LV myocardium at a frame rate between 50 and 80 frames/second. Echocardiograms suitable for analysis were included when the patient was free from biopsy confirmed ACR at 1-year after transplantation.

Systolic strain, diastolic strain rate and mechanical dispersion time to peak strain parameters were determined via 2D speckle tracking analysis using a vendor independent system (Image arena version 4.6.4, TomTec imaging systems, Unterschleißheim, Germany). Strain was quantified using the 2D cardiac performance analysis package, which required manual contouring of the endocardial border in the apical 4-chamber, apical 2-chamber, apical long-axis, and focused RV view obtained from the apical window. Determination of peak LV strain was measured at the nadir of the peak negative deformation strain value from all regional LV strain curves and averaged for all 16 myocardial segments and expressed in absolute values. End systole was determined at the time of aortic valve closure (AVC). Timing of AVC was defined as the time from the onset of the R-wave on the ECG to the termination of the left ventricular outflow tract signal by pulsed wave Doppler. Determination of peak systolic RV strain was measured using peak deformation as seen on the segmental strain curve. LV GLS was calculated as an average peak strain from all 16 myocardial segments and expressed in absolute values. Systolic mechanical dispersion was calculated as the standard deviation of the time from R-wave to peak systolic strain in all 16 segments. RV GLS was calculated as an average peak strain from 3 free wall RV myocardial segments. Systolic mechanical dispersion for the RV was calculated as the standard deviation of the time from R-wave to peak systolic strain in 3 free wall myocardial segments. Strain measurements are expressed in absolute values. Diastolic speckle tracking analysis was performed from the same apical images of the LV and RV used for GLS. Longitudinal diastolic strain rate, which is defined as the rate of deformation in percent of strain per second during diastole (1/s), was calculated from an average of the 18 LV myocardial segments during early diastole. LV early diastolic strain rate was measured during the peak positive signal waveform after systole and before the P-wave on the ECG as previously described[12]. RV early diastolic strain rate (RV ESr) was measured from the 3 RV free wall myocardial segments in the same manner as for the LV.

### Statistical analysis

Normal distribution for the continuous data was verified using the Shapiro-Wilk test. Continuous data is expressed as mean values ± standard deviation. Comparison of the continuous data was performed by an ANOVA with the Bonferroni correction for multiple comparisons. A univariate binary linear logistic regression model analysis was performed when comparing continuous variables with outcome data of cardiovascular morbidity and all-cause mortality whilst a multivariate Cox proportional hazard regression model was used to correct for blood pressure, CAV, de-novo regional wall motion abnormalities on stress echo, LV EF and admission with heart failure. For optimal determination of sensitivity and specificity, a value closest to the upper left corner of the receiver operator characteristic (ROC) curve was chosen to define the optimal cutoff values for Kaplan-Meier analysis. Odds ratios (OR) and hazard ratios (HR) with 95% confidence intervals (CI) were calculated and reported and significance was set at p < 0.05.

Ten patients were randomly selected and remeasured by two observers (R.C. and N.E.) who were blinded to the clinical data and each other’s results. Intra-observer variability was performed by R.C. 3 months after the original strain analysis. Inter-observer variability was performed by N.E. repeating measurements from the same images. Intra- and inter-observer variabilities were calculated by intraclass correlation coefficient (ICC) and the standard error of measurement (SEM). All statistical analysis was performed using IBM SPSS Statistics 25 (Chicago, IL, USA).

## Results

There were 128 heart transplant recipients who satisfied inclusion criteria and survived the first-year post transplantation were included in this study. No patients had evidence of CAV by coronary angiography, ACR or antibody mediated rejection from endomyocardial biopsy at the time of strain analysis performed at 1-year post-transplantation. At the 6-year post-transplantation period, 21 patients (17%) died, 47 patients (37%) developed CAV, 7 patients (6%) required percutaneous or surgical revascularisation, 14 patients (11%) experienced ≥ 2R ACR, 7 patients (6%) experienced antibody mediated rejection, 17 patients (13%) developed arrhythmia requiring treatment and 31 patients (24%) were hospitalised with treatment requiring heart failure. Table 1 displays demographics acquired at 1-year post transplantation for the patients in the three rejection groups. There were no significant differences between the groups for patient age, donor age, ischaemic time, blood pressure and comorbidities (diabetes and hypertension). There was a significant increase in patients taking Prednisolone in RS group 3 (57% in group 3 vs. 23% in group 2, p = 0.023) and calcium channel blockers in RS group 3 (29% in group 3 vs. 49% and 47% for groups 1 and 2 respectively, p = 0.043). Table 2 shows various standard and strain echocardiographic parameters for the 3 RS groups with post-hoc analysis for significant results.

**Table 1:** Patient characteristics according to Rejection Score Group

Variable	Rejection Score 1 (n = 43)	Rejection Score 2 (n = 64)	Rejection Score 3 (n = 21)	ANOVA, p
Patient Age (years)	49 ± 14.8	47 ± 16.3	48 ± 14.1	0.441
Donor Age (years)	39 ± 12.4	37 ± 14.9	38 ± 14.9	0.596
Gender mismatch	No (89%)	No (81%)	No (76%)	0.358
Ischaemic time (minutes)	202 ± 99	224 ± 94	257 ± 105	0.092
**Reason for Transplantation**				
*Cardiomyopathy*	19 (43%)	35 (54%)	7 (33%)	-----
*Ischaemic Heart Disease*	18 (41%)	28 (43%)	12 (62%)	-----
*Congenital Heart Disease*	3 (7%)	2 (3%)	0 (0%)	-----
*Other*	3 (7%)	0 (0%)	1 (5%)	-----
Height (cm)	175 ± 9.1	171 ± 9.8	173 ± 10.7	0.921
Weight (kg)	82 ± 15.6	78 ± 17.2	84 ± 14.9	0.340
BSA (m²)	1.99 ± 0.22	1.91 ± 0.25	2.00 ± 0.22	0.243
Systolic Blood Pressure (mmHg)	133 ± 14	135 ± 17	133 ± 18	0.874
Diastolic Blood Pressure (mmHg)	79 ± 11	79 ± 9	78 ± 15	0.897
Diabetes (%)	3 (7%)	6 (9%)	3 (14%)	0.741
Hypertension (%)	17 (39%)	26 (41%)	8 (38%)	0.441
**Medications**				
*Prednisolone*	10 (23%)	15 (23%)	12 (57%)	0.023
*Cyclosporine*	14 (33%)	29 (45%)	13 (62%)	0.094
*Tacrolimus*	19 (44%)	31 (48%)	11 (52%)	0.378
*Mycophenalate*	22 (51%)	44 (69%)	14 (67%)	0.663
*Everolimus*	13 (30%)	23 (36%)	5 (24%)	0.874
*Sirolimus*	2 (5%)	5 (8%)	2 (10%)	0.996
*Statins*	31 (72%)	51 (80%)	18 (86%)	0.236
*ACE / AT 2 inihibitor*	33 (77%)	25 (39%)	14 (67%)	0.089
*Frusemide*	9 (21%)	8 (13%)	6 (29%)	0.128
*Thiazide*	7 (16%)	9 (14%)	4 (19%)	0.563
*Calcium Channel Blocker*	21 (49%)	30 (47%)	6 (29%)	0.043
*Beta Blocker*	10 (23%)	10 (16%)	5 (24%)	0.781
**Biochemistry**				
*Creatinine (µmol/L)*	126 ± 42.4	161 ± 31.1	188 ± 29.4	0.071
*Haemoglobin (g/dl)*	11.8 ± 2.2	10.4 ± 1.7	10.1 ± 2.1	0.279

*BSA: Body Surface Area; ACE: Angtiotensin Converting Enzyme; AT2; Angiotensin Two Receptor Blocker*

**Table 2:** Echocardiographic parameters by Rejection Score Group

					Post-hoc analysis, p-value		
**Variable**	**Rejection Score 1 (n = 43)**	**Rejection Score 2 (n = 64)**	**Rejection Score 3 (n = 21)**	**ANOVA, p**	**RS 1 vs. RS 2**	**RS 1 vs. RS 3**	**RS 2 vs. RS 3**
LV EF (%)	62 ± 8	63 ± 6	64 ± 7	0.577	-	-	-
LV Diastolic Volume (mL)	112 ± 22	103 ± 32	104 ± 32	0.470	-	-	-
LV Systolic Volume (mL)	42 ± 11	38 ± 12	39 ± 16	0.480	-	-	-
LV End-Diastolic Dimension (cm)	4.8 ± 1.1	4.5 ± 0.5	4.8 ± 0.5	0.313	-	-	-
LV End-Systolic Dimension (cm)	3.2 ± 0.8	3.0 ± 0.6	3.1 ± 0.5	0.678	-	-	-
Interventricular Septum Dimension (cm)	1.1 ± 0.2	1.1 ± 0.2	1.1 ± 0.3	0.860	-	-	-
Posterior Wall Dimension (cm)	0.9 ± 0.2	1.0 ± 0.2	1.1 ± 0.2	0.078	-	-	-
E-Velocity (cm/s)	87 ± 24	84 ± 22	90 ± 18	0.688	-	-	-
Septal e’ (cm/s)	9 ± 2	8 ± 2	7 ± 3	0.109	-	-	-
Lateral e’ (cm/s)	14 ± 3	12 ± 3	11 ± 3	0.069	-	-	-
Averaged e’ (cm/s)	11 ± 2	11 ± 2	9 ± 3	0.096	-	-	-
E/e’	8 ± 3	9 ± 3	12 ± 7	0.077	-	-	-
RV Fractional Area Change (%)	44 ± 6	45 ± 11	35 ± 11	0.467	-	-	-
RV TAPSE (cm)	1.5 ± 0.5	1.5 ± 0.3	1.8 ± 0.1	0.694	-	-	-
RV S’ (cm/s)	9 ± 3	11 ± 3	11 ± 3	0.296	-	-	-
RV IVRT (ms)	54 ± 6	47 ± 13	39 ± 19	0.282	-	-	-
TR Velocity (m/s)	2.1 ± 0.5	2.3 ± 0.4	2.5 ± 0.1	0.501	-	-	-
RVSP (mmHg)	22 ± 7	29 ± 16	32 ± 3	0.516	-	-	-
LV GLS (%)	18.1 ± 2.1	15.7 ± 3.1	15.3 ± 3.2	0.0001	0.0001	0.0003	1.0000
LV ESr (1/s)	1.05 ± 0.27	0.92 ± 0.27	0.88 ± 0.27	0.024	0.046	0.024	1.000
LV Systolic Mechanical Dispersion (ms)	47 ± 21	55 ± 32	59 ± 18	0.216	-	-	-
RV GLS (%)	23.1 ± 4.9	19.6 ± 4.5	16.9 ± 1.9	0.0001	0.003	0.0001	0.0021
RV ESr (1/s)	1.25 ± 0.33	0.97 ± 0.28	0.91 ± 0.2	0.003	0.002	0.003	0.922
RV Systolic Mechanical Dispersion (ms)	44 ± 19	57 ± 36	56 ± 15	0.031	0.019	0.014	1.000

*TAPSE: Tricuspid Annular Plane Systolic Excursion; IVRT: Isovolumic Relaxation Time; TR Velocity: Tricuspid Regurgitant Velocity; RVSP: Right Ventricular Systolic Pressure. LV volumetric analysis was performed by Simpson’s method*

### Primary Endpoint

Tables 3 and 4 demonstrate univariate binary linear logistic regression and multivariate Cox proportional hazard regression analyses for primary and secondary endpoint data.

### Univariate Analysis

There were significant relationships between echocardiographic and clinical variables for 6-year all-cause mortality (Table 3). LV GLS, LV ESr, RV ESr, an admission with symptoms consistent with heart failure and development of CAV demonstrated the highest significant odds ratios. Significant odds ratios were also seen for RV GLS, ischaemic time, and the presence of hypertension.

### Multivariate Analysis

**Fig. 1:**
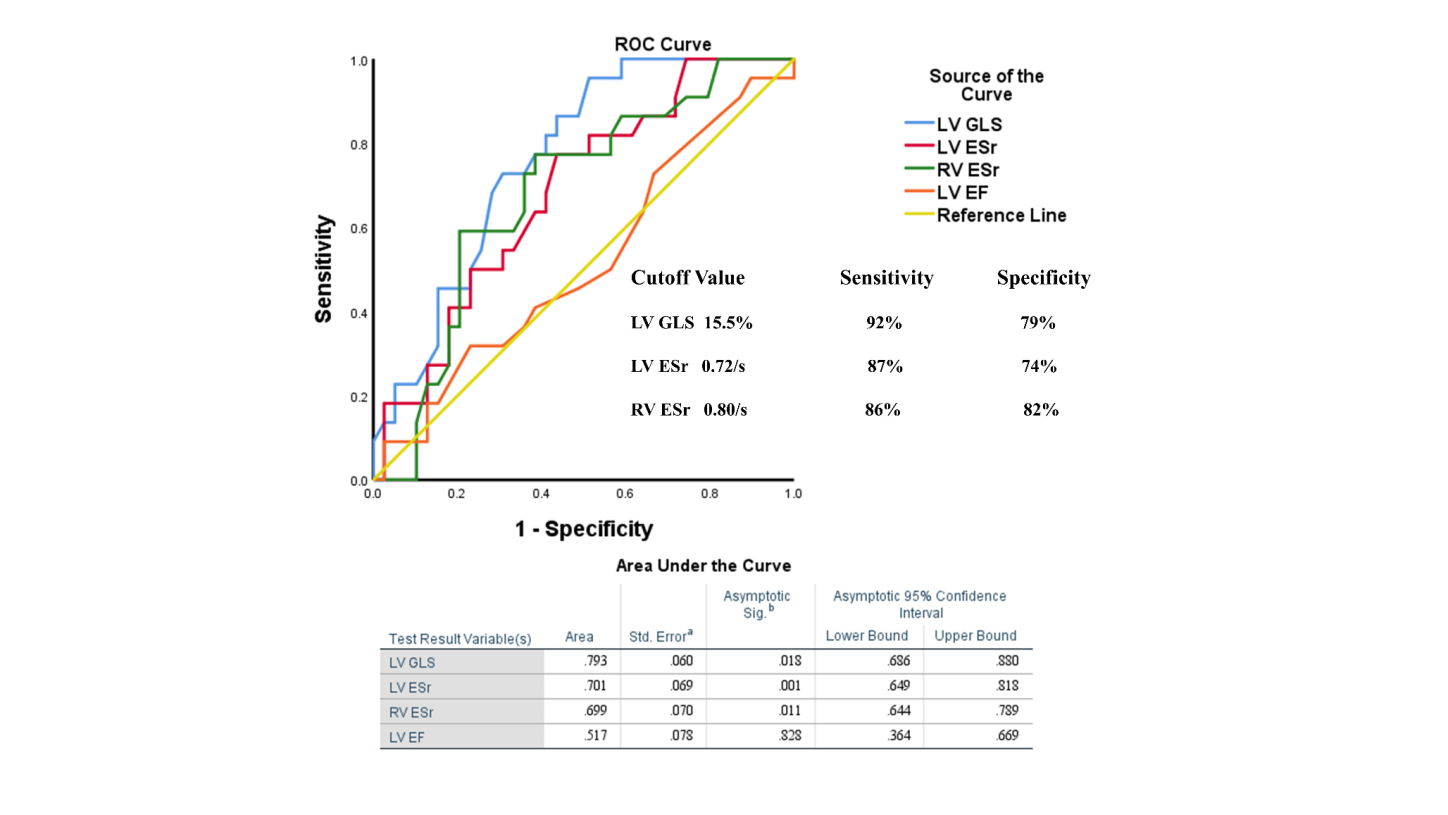
Forest plot detailing hazard ratios for predictors of 6-year all-cause mortality from multivariate Cox proportional hazard regression analysis corrected for blood pressure, CAV, de-novo regional wall motion abnormalities on stress echo, LV EF and admission with heart failure. LV GLS: Left Ventricular Global Longitudinal Strain, LV ESr: Left Ventricular Early Strain rate, RV GLS: Right Ventricular Global Longitudinal Strain, RV ESr: Right Ventricular Early Strain rate

**Fig. 2:**
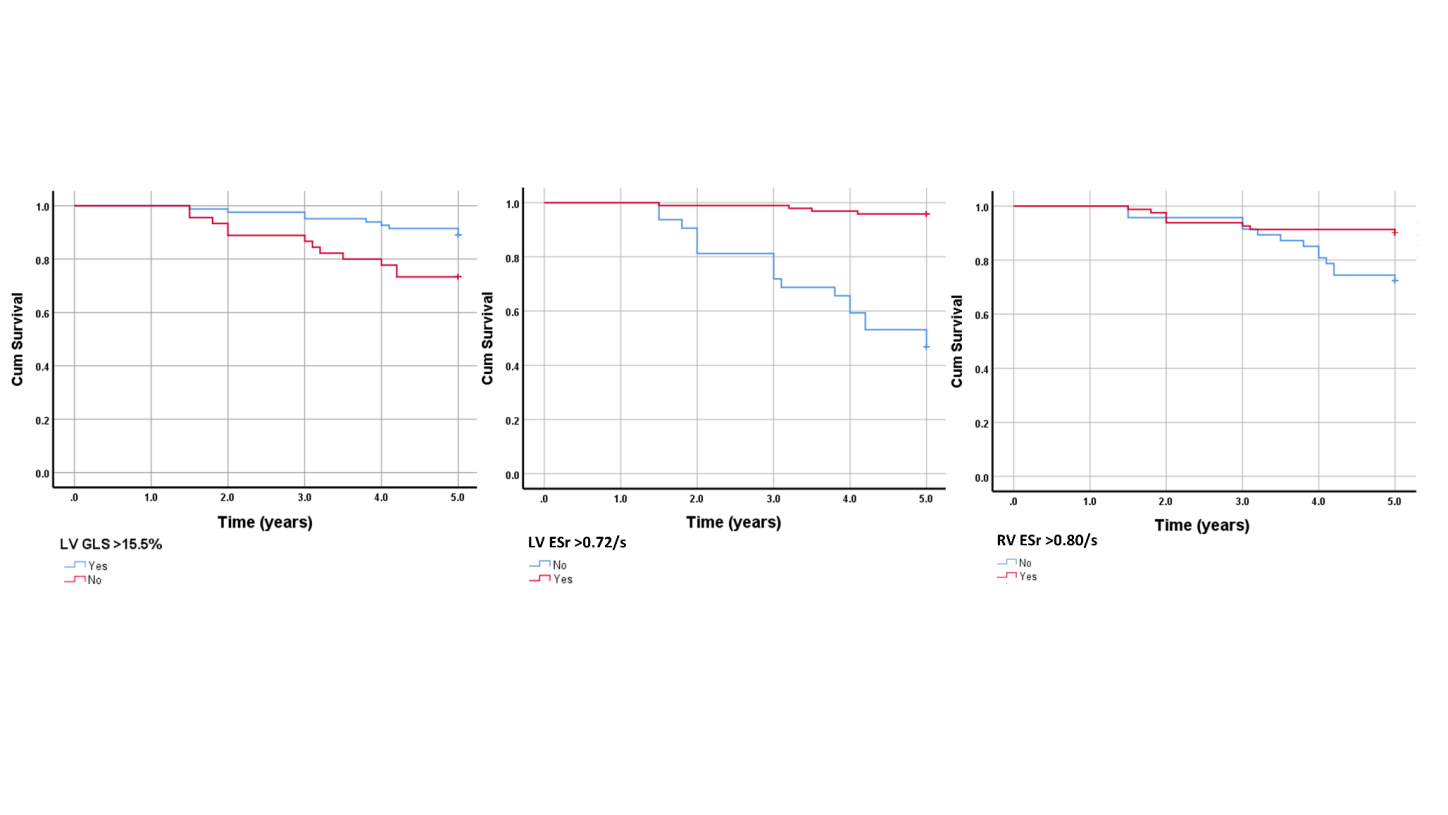
ROC curves for all-cause mortality for LV GLS, LV ESr, RV ESr, and LV EF. LV GLS: Left Ventricular Global Longitudinal Strain, LV ESr: Left Ventricular Early Strain rate, RV ESr: Right Ventricular Early Strain rate, LV EF: Left Ventricular Ejection Fraction

**Fig. 3:**
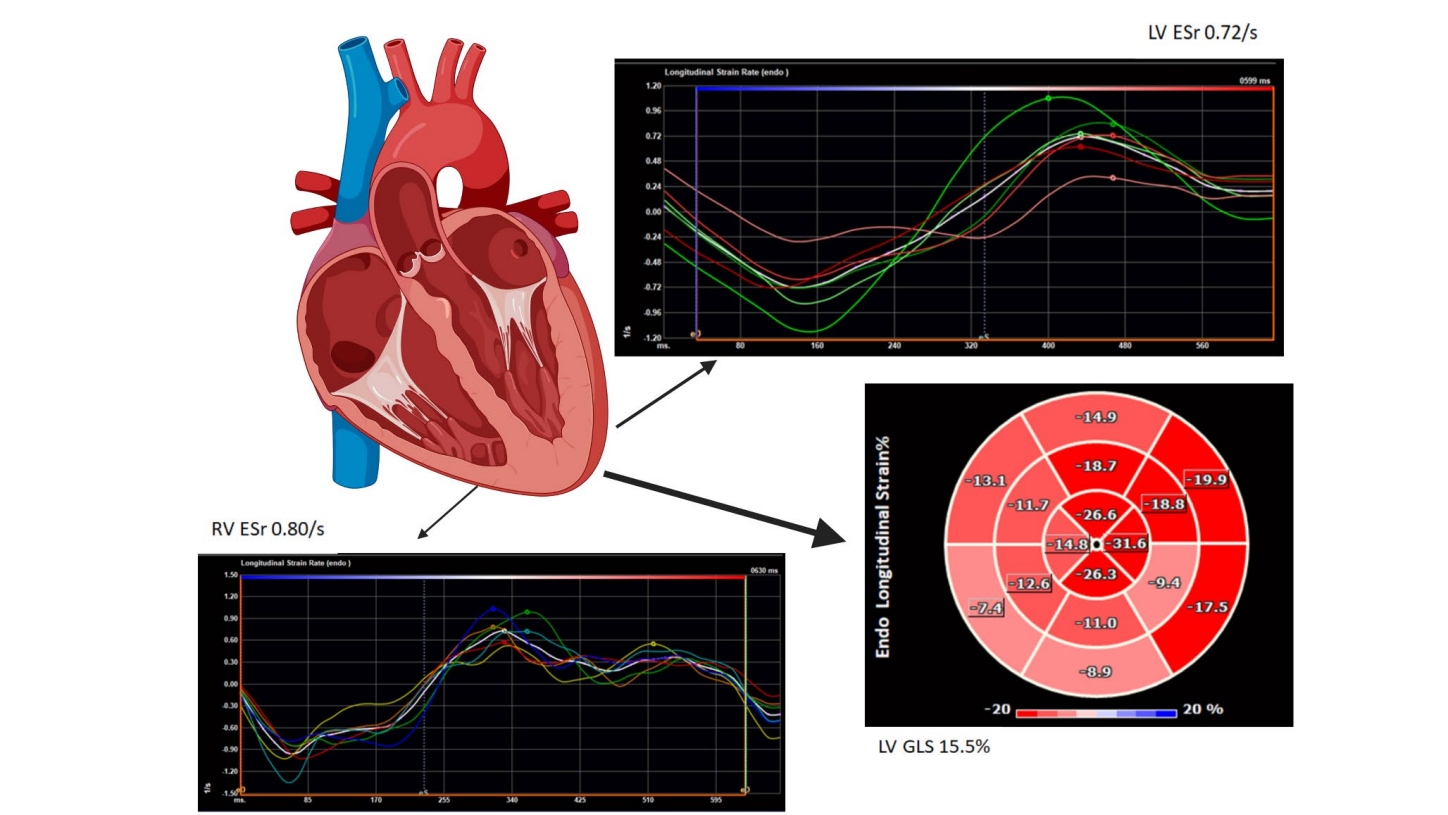
Kaplan-Meier plots demonstrating survival from 6-year all-cause mortality. Abbreviations: LV GLS: Left Ventricular Global Longitudinal Strain, LV ESr: Left Ventricular Early Strain rate, RV ESr: Right Ventricular Early Strain rate

**Fig. 4:**
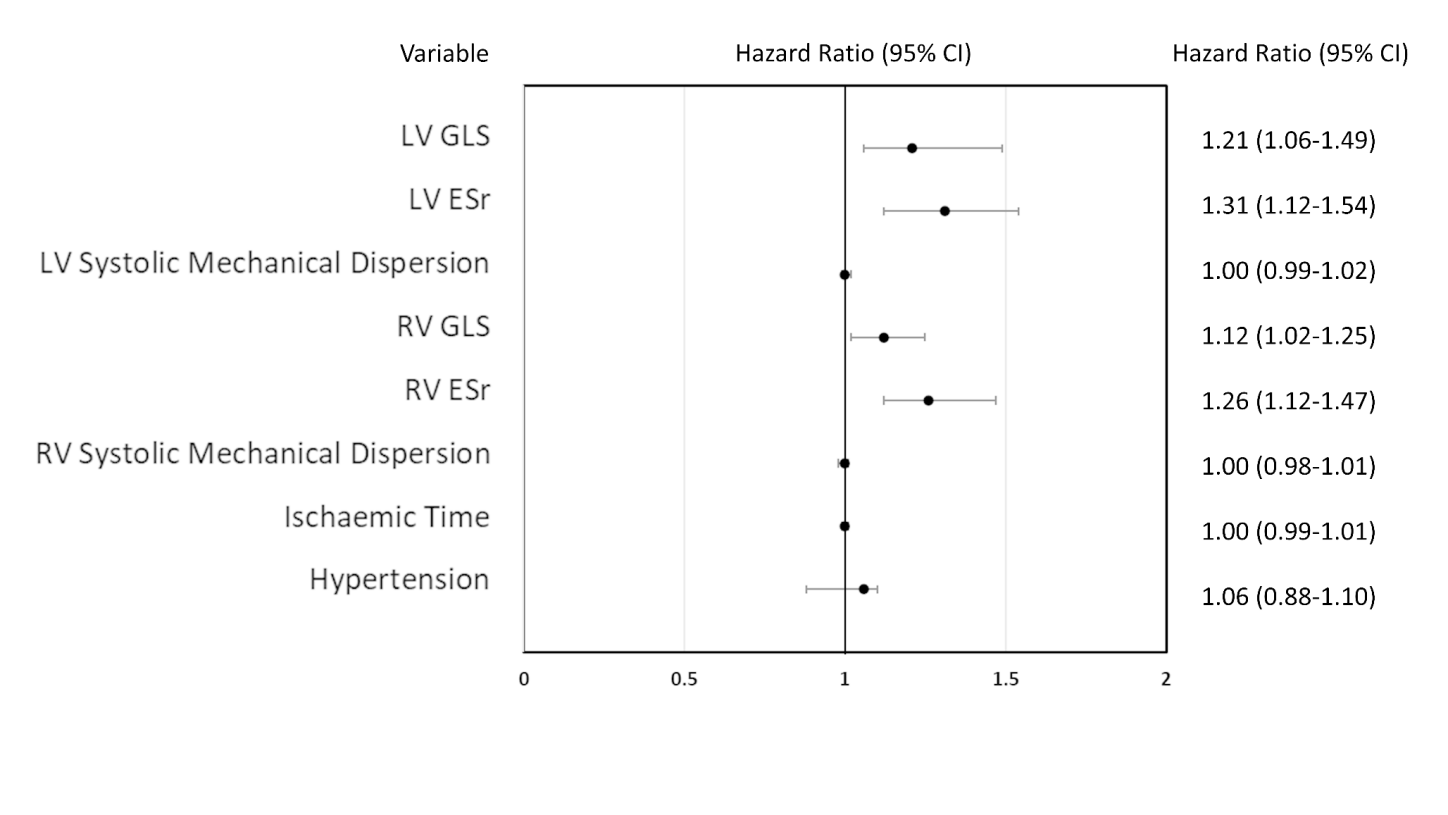
Diagrammatical representation of LV GLS, LV ESr and RV ESr strain analysis with relevant cutoff values determined for survival analysis. LV GLS: Left Ventricular Global Longitudinal Strain, LV ESr: Left Ventricular Early Strain rate, RV ESr: Right Ventricular Early Strain rate

Cox proportional hazard multivariate regression analysis revealed LV GLS, LV ESr, RV GLS and RV ESr to be significant predictors of 6-year all-cause mortality (Fig. 1). Figure 2 shows sensitivity and specificity values from ROC curve analysis to determine appropriate cutoff values for Kaplan-Meier survival analysis. Kaplan-Meier plots demonstrating all-cause mortality survival revealed LV GLS > 15.5% (log-rank test, p = 0.018), LV ESr > 0.72/s (log-rank test, p = 0.001) and RV ESr > 0.80/s (log-rank test, p = 0.011) showed significant survival outcomes (Fig. 3). Figure 4 shows examples of strain parameters and their cutoff values for survival analysis.

**Table 3:** Univariate and Multivariate analysis for 6-year all-cause mortality

Variable	Univariate Analysis	P-value	Multivariate Analysis	P-value
	**Odds ratio (95% CI)**		**Hazard ratio (95% CI)**	
LV GLS	1.23 (1.04–1.44)	0.01	1.21 (1.06–1.49)	0.04
LV ESr	3.91 (2.14–4.76)	0.02	1.31 (1.12–1.54)	0.001
LV Systolic Mechanical Dispersion	0.98 (0.97–1.00)	0.06	1.00 (0.99–1.02)	0.32
RV GLS	1.11 (0.99–1.23)	0.047	1.12 (1.02–1.25)	0.04
RV ESr	3.17 (2.01–4.55)	0.02	1.26 (1.12–1.47)	0.001
RV Systolic Mechanical Dispersion	0.98 (0.97–1.00)	0.06	1.00 (0.98–1.01)	0.75
Ischaemic Time	0.99 (0.98–0.99)	0.02	1.00 (0.99–1.01)	0.06
Hypertension	0.29 (0.093–0.93)	0.03	1.06 (0.88–1.10)	0.08
Systolic BP	0.98 (0.95–1.02)	0.53		
Diastolic BP	0.97 (0.91–1.03)	0.97		
Weight	0.99 (0.96–1.02)	0.77		
CAV	2.70 (1.04–7.02)	0.04		
De-Novo RWMA on Stress Echo	3.38 (1.97–5.21)	0.06		
Max HR on Stress Echo	1.02 (0.99–1.05)	0.17		
METs on Stress Echo	1.01 (0.83–1.23)	0.85		
Admission with Heart Failure	4.89 (3.69–8.26)	0.0001		
LVEF	0.98 (0.90–1.05)	0.59		
RV FAC	0.92 (0.83–1.02)	0.159		

*BP: Blood Pressure, RWMA: Regional Wall Motion Abnormality, MET: HR: Heart Rate, Metabolic Equivalence of Task, RV FAC: Right Ventricular Fractional Area Change. Multivariate analysis corrected for blood pressure, CAV, de-novo regional wall motion abnormalities on stress echo, LV EF and admission with heart failure*

### Secondary Endpoint

### Univariate Analysis

Binary regression analysis for secondary endpoint data showed LV GLS, LV ESr and RV ESr to be all significant predictors of morbidity after the first-year post transplantation (Table 4).

### Multivariate Analysis

Cox proportional hazard multivariate regression analysis revealed LV GLS, LV ESr and RV ESr were significant predictors of morbidity.

The reproducibility of strain measurements was excellent as seen in Table 5.

**Table 4:** Univariate and Multivariate analysis for morbidity data

Variable	Univariate Analysis	P-value	Multivariate Analysis	P-value
	**Odds ratio (95% CI)**		**Hazard ratio (95% CI)**	
LV GLS	0.82 (0.72–0.93)	0.003	1.113 (1.092–1.234)	0.019
LV ESr	0.77 (0.56–0.92)	0.001	1.212 (1.091–1.394)	0.024
LV Systolic Mechanical Dispersion	0.995 (0.96–1.03)	0.760	1.001 (0.991–1.011)	0.819
RV GLS	0.732 (0.518–1.036)	0.078	0.906 (0.772–1.062)	0.223
RV ESr	1.89 (1.66–2.12)	0.001	1.299 (1.151–1.424)	0.011
RV Systolic Mechanical Dispersion	1.002 (0.974–1.030)	0.910	1.009 (0.999–1.018)	0.073
Ischaemic Time	1.012 (0.990–1.034)	0.298		
Systolic BP	0.989 (0.955–1.025)	0.543		
Diastolic BP	1.048 (0.920–1.193)	0.485		
Weight	0.964 (0.563–1.650)	0.964		
Max HR on Stress Echo	0.976 (0.897–1.063)	0.581		
METs on Stress Echo	0.804 (0.457–1.414)	0.448		
LVEF	0.872 (0.685–1.110)	0.266		
RV FAC	0.897 (0.696–1.155)	0.399		

*BP: Blood Pressure, MET: HR: Heart Rate, Metabolic Equivalence of Task, LVEF: Left Ventricular Ejection Fraction, RV FAC: Right Ventricular Fractional Area Change. Morbidity data consisted of secondary endpoints. Multivariate analysis corrected for blood pressure, CAV, de-novo regional wall motion abnormalities on stress echo, LV EF and admission with heart failure*

**Table 5:** Inter- and Intraobserver variability

	Intra-observer	Inter-observer
**Variable**	**ICC (95% CI)**	**ICC (95% ICI)**
LV GLS	0.94 (0.87–0.99)	0.98 (0.96–0.99)
LV ESr	0.98 (0.95–0.99)	0.95 (0.91–0.99)
LV systolic mechanical dispersion	0.96 (0.82–0.99)	0.97 (0.86–0.99)
RV GLS	0.93 (0.91–0.96)	0.91 (0.89–0.93)
RV ESr	0.95 (0.93–0.98)	0.92 (0.90–0.94)
RV systolic mechanical dispersion	0.93 (0.92–0.95)	0.94 (0.92–0.96)

*ICC: Intraclass correlation; CI: Confidence intervals*

## Discussion

*Highlights*.

- LV GLS is a significant predictor of both primary and secondary outcome with 15.5% being the best predictor of 6-year all-cause mortality in heart transplant patients.
- RV GLS is a significant predictor of 6-year all-cause mortality by multivariate and univariate analyses.
- Left and right ventricular diastolic strain rate are also good predictors of both all-cause mortality and complications associated post-heart transplantation.
- Unlike novel strain parameters of myocardial function, traditional 2D and Doppler parameters of systolic and diastolic function showed no significant differences between rejection score groups.

The main finding of this study highlights repeated episodes of ACR, determined by enodomyocardial biopsy during the first-year post heart transplantation results in reduced longitudinal systolic and diastolic function as measured by 2D speckle tracking strain analysis. In contrast, traditional 2D and Doppler parameters of systolic and diastolic function showed no significant differences between RS groups. These results highlight the profound effect strain analysis can play in the assessment of cardiac allograft function.

The results of this study clearly show that repeated episodes of ACR can result in significant reductions in systolic LV and RV longitudinal strain with LV GLS varying from 18.1% in RS group 1 (i.e., patients that experienced the mildest burden of rejection) reducing to 15.3% in those with severe burden of rejection in this cohort (RS group 3). Novel diastolic strain rate parameters for the LV and RV were also significantly reduced in line with rejection severity underscoring the importance of investigating diastolic strain rate parameters post-transplantation[12, 21]. Normal values for strain have been investigated post cardiac transplantation yielding varied results for LV GLS ranging from 13.4%[22] to 20.0%[23] and RV GLS ranging from 16.9%[24] to 26.9%[23] highlighting the heterogeneity of these strain parameters in this population of patients. Such heterogeneity seen could be attributed to a number of factors that are known to reduce longitudinal strain such as the surgical procedure, ischaemia-reperfusion injury, LV remodeling, hypertension, fibrosis due to immunosuppressive therapy, macro and micro-vascular perfusion and increased LV-preload[25–30]. Nevertheless our study shows that reduction of LV and RV strain parameters may be immune mediated in accordance with a higher burden of ACR which is in-keeping with previously published results[18].

After adjusting for potential confounders, the present study demonstrated a significant correlation between both systolic (LV and RV GLS) and diastolic (LV ESr and RV ESr) and all-cause mortality. A cutoff value for LV GLS of 15.5% was a significant and strong predictor of mortality which is concordant with other reported work in patients post heart transplant and asymptomatic aortic stenosis[19, 31]. Interestingly, RS groups 2 and 3 who experienced moderate and severe rejection burden had LV GLS values of 15.7% and 15.2% respectively. Of note, the rejection score was not corrected for in the multivariate regression analysis as it isn’t considered an established risk stratification parameter within the clinical assessment of rejection burden. It has been previously reported that high rejection burden increases the development of CAV suggesting that an immune-mediated response is at play[2] and longitudinal myocardial function depends on the micro and macrovascular system. Studies have demonstrated a common occurrence of microvascular dysfunction in heart transplant patients triggered by repeated episodes of ACR and patients with severe or repeated episodes of ACR are likely to receive higher doses of immunosuppression which is associated with myocardial fibrosis [25, 28] and impaired longitudinal myocardial function with GLS being correlated with CAV[11]. Even though patients in the current study were free from CAV at the time of strain analysis, development of CAV was a strong predictor of the primary end point in univariate analysis.

The prognostic importance of LV GLS as a marker of outcome has been reported in heart transplant patients during the first-year post-transplant[15] and at an intermediate phase of 2-years post-transplant[16] but extension to longer term outcome results are scarce, especially investigating RV strain.

The utility of RV strain to detect ACR has been proven to be useful[13, 21] but its prognostic role in predicting adverse outcomes has yet to be fully explored. The current study showed significant differences in RV GLS between RS groups. Furthermore, RV GLS was a significant predictor of the primary endpoint. Barakat et al[14] investigated the prognostic value of RV GLS at one year with 5-year follow up. Results showed RV GLS of 17.2% was impaired in stable heart transplant patients analysed at 1-year and was further reduced to 14.6% in patients who subsequently experienced endpoints associated with post-transplant morbidity outcomes. A cutoff value of 16.4% was associated with morbidity outcomes over 5-year follow up. These findings could not be replicated in the current study however a similar RV GLS value of 16.9% was observed in the RS 3 group who experienced the highest burden of ACR. Traditional 2D and Doppler assessment of RV function showed no significant differences between RS groups, as with the LV, indicating that these indices lack the sensitivity to detect significant changes in cardiac function associated with ACR burden.

Recently, Clemmensen et al[19] showed that a composite model of LV GLS and Doppler based diastolic filling pressure parameters were stronger predictors of cardiovascular outcomes in heart transplant patients, while diastolic filling pressures alone were not significant predictors of outcome. In contrast, the use of diastolic strain rate has provided utility in determining prognostic information surrounding cardiovascular mortality in the general population[32], in patients with type 2 diabetes[33], heart failure[34] and patients with acute coronary syndrome[35]. In the current study the assessment of LV ESr, which is a novel strain parameter equivalent to LV diastolic function, was another strong predictor of the primary and secondary end points of cardiovascular morbidity and all-cause mortality. It is well known that ACR is associated with local oedema, mononuclear lymphocyte infiltration, myocyte damage and development of myocardial fibrosis[3, 17]. The process surrounding fibrosis often effects the subendocardial myocardial fibers which leads to reduced longitudinal myocardial function[9, 36] measured using strain in stable transplant patients and allograft failure[10–13].

Interestingly, RV ESr was also a significant predictor for both the primary and secondary endpoints in univariate and multivariate analysis with a cut of value for RV ESr of 0.80/s. This is the first study to date to investigate novel diastolic strain rate RV ESr and it can be clearly seen that RV ESr is of value determining the outcome of transplant patients.

### Clinical Implications

The results of the present study clearly show that a single assessment of myocardial strain performed on the LV and RV after transplantation might be a useful noninvasive tool in helping identify heart transplant patients who are subject to a poor clinical prognosis. Although echocardiography is used in the serial follow up assessment of heart transplant patients, traditional echo-based parameters may not be sufficiently sensitive in capturing the spectrum of rejection severity or provide predictive outcomes. In contrast, echocardiographic assessment using strain analysis may provide early prognostic outcomes that can be used to inform and optimise treatment strategies that will improve short and longer-term patient outcomes. Furthermore, strain assessment should not be limited to a single point in time as serial assessment of myocardial strain can provide useful insights into the patient specific changes associated with ACR and CAV[11, 12, 37].

## Limitations

This study was a single center retrospective observational design from a tertiary referral center including selection bias determining a moderate sample size with a modest event rate. Radial and circumferential strain measures were not performed due to evidence of reduced reproducibility for these strain parameters. Microvascular perfusion could not be ascertained which may provide relevant information regarding impairment of long axis function in this population.

## Conclusions

Amongst heart transplant recipients, recurrent rejection burden at 1-year is associated with reduction of LV and RV strain parameters and negatively impact medium term survival. Traditional 2D and Doppler based parameters of cardiac function post-transplant are inferior compared to strain assessment of LV and RV function. LV GLS and LV ESr are the best predictors of mortality outcome at 6 years post transplantation.
